# Comparison of Respiratory-Triggered (RT) 3D cine steady-state free precession cardiac mri with standard 2D cine imaging and magnetic resonance angiography in Congenital Heart Disease (CHD)

**DOI:** 10.1186/1532-429X-17-S1-P29

**Published:** 2015-02-03

**Authors:** Lamya A Atweh, Karen Lyons, Ramkumar Krishnamurthy, Amol Pednekar, Rajesh Krishnamurthy

**Affiliations:** 1 Diagnostic Radiology, American University of Beirut Medical Center, Beirut, Lebanon; 2 EB Singleton Department of Pediatric Radiology, Texas Children's Hospital, Houston, TX, USA; 3 Philips Healthcare, Houston, TX, USA

## Background

This is a pilot study to evaluate the performance of whole-heart isotropic RT-3D cine SSFP sequence as an efficient single solution for assessment of morphology and function in CHD by comparing it to: 1. Short axis (SA) 2D cine SSFP for LV volumetry and function 2. Multiplanar 2D cine SSFP for intracardiac morphology 3. Cine 2D SSFP and gadolinium (Gd)-enhanced 3D MRA for aortic root size 4. Gd-3D MRA for vessel diameter (VD) 5. Gd-3D MRA, and cine 2D SSFP for morphology of extracardiac vasculature 6. Gd-3D MRA and multiplanar cine 2D SSFP for overall diagnosis.

## Methods

15 patients with CHD (8M, mean age 11.7 years), referred for clinic CMR (80% sedated) underwent an additional RT 3D SSFP sequence (Table 1). LV volumetry was performed on the 3D and 2D cine SSFP SA datasets by the same reader on CMR42 software. VD measurements, image quality analysis, and pathologic findings were assessed on 3D, 2D SSFP and MRA datasets using Vitrea^TM^ by another blinded reader. Pearson and Spearman rank correlation coefficients were calculated and paired t tests performed to compare volumetry, imaging scores, maximal VD, and diagnostic accuracy.

**Table 1:** Respiratory Triggered 3-D Cine SSFP Technique and Grading Methodology

Duration	4.5-7 minutes
Hardware	Phillips Achieva 1.5T magnet; 5-channel phased array coil; Max. parallel acceleration: 2 (2D); 2X1.5 (3D)

Acquisition Parameters	Axial or sagittal acquisition; respiratory triggered, retrospective EKG gated, TR/TE/α = 3/1.5/60; voxel size 1.5-2.5; SENSE 2.6-3.6; mean temporal resolution = 35 ms

Imaging Targets & Grading Methodology	Quantitative LV volumetry (3D vs 2D cine SSFP): LVEDV, LVESV, LVEF Vessel Measurements (3D cine SSFP vs 2D cine SSFP and Gd-3D MRA): Maximal intersinus aortic root, ascending aorta, aortic isthmus, MPA and LPA diameter Cardiac Morphology Qualitative scoring (3D vs 2D cine SSFP): Atrial morphology/ function (0-8): Edge definition (0-2), Blood myocardial contrast (0-2), Atrial septum (0-2), Crista terminalis (0-2), Aortic valve morphology/ function (0-8): Annulus (0-2), Number of leaflets (0-2), Valve motion (0-2), Valve coaptation (0-2) Tricuspid valve morphology/ function (0-10): Annulus (0-2), Number of leaflets (0-2), Valve motion (0-2), Valve coaptation (0-2), Suspensory apparatus (0-2) Left Ventricular morphology (0-15): Edge definition (0-5), Blood myocardial contrast (0-5), Interslice alignment (0-5) Extra-cardiac Vasculature Qualitative Scoring (3D cine SSFP vs Gd-3D MRA): Aorta (0-8): Edge definition (0-2), Blood pool homogeneity (0-2), First order branches (0-2), Second order branches (0-2) Pulmonary arteries (0-10): Edge definition (0-2), Blood pool homogeneity (0-2), First order branches (0-2), Second order branches (0-2), Arteriovenous separation (0-2) Pulmonary veins (0-10): Edge definition (0-2), Blood pool homogeneity (0-2), First order branches (0-2), Second order branches (0-2), Arteriovenous separation (0-2) SVC/ Azygous vein (0-8): Edge definition (0-2), Blood pool homogeneity (0-2), First order branches (0-2), Second order branches (0-2)

## Results

**Figure 1:**
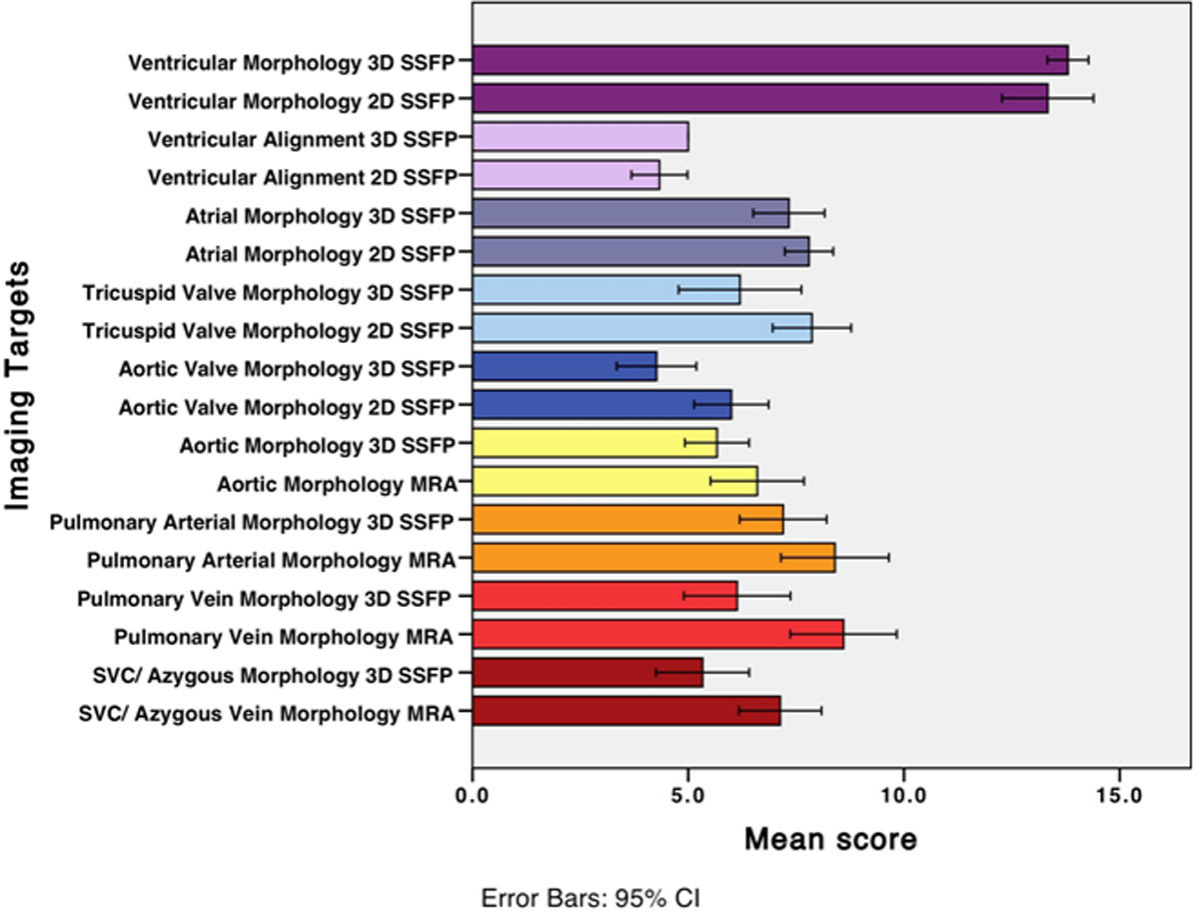
Results comparison of RT-3D SSFP with conventional CMR sequences for morphology and morphology and function in CHD

1. Excellent correlation between LV volumetry from 3D SSFP and 2D SSFP (r=0.91-0.99, p<.0001). Only LVESV was significantly different (mean diff -1.63 cc, p 0.03). 2. Excellent correlation between aortic root measurements on 3D and 2D SSFP, and on 3D SSFP and MRA (r=0.98, 0.96, p<0.0001). Maximal intersinus root diameter was not significantly different. 3. Excellent correlation between VD on 3D SSFP and MRA (r=0.96-0.99, p<0.001). Only MPA diameter was significantly different (mean diff -1.92 mm, p 0.01). 4. Moderate performance for intracardiac morphology and pathology; 3D scores of aortic and tricuspid valves were lower than 2D (mean diff -28.9%, -21.2%, p=0.003, 0.005). Interslice alignment was better on 3D imaging (mean diff 8.9%, p=0.04). 5. There was no significant difference in the overall aorta and PA scores; but 3D cine SSFP scores were lower for evaluation of 1st and 2nd order vessel branches, and for AV separation (mean diff -23%, -53%, -27%, p=0.025, <0.0001, 0.01). 3D scores were also lower for the veins (mean diff -25%, -29% p=0.03, 0.01). 6. The overall diagnosis was different (p=0.02) in 5/15 cases (9 missed findings) on 3D imaging. 5/9 reflected valve pathology, 2 evaluation of 1st order vessels, 1 of aorta, and 1 of atrial septum. 4 of the missed findings were partly due to suppression of turbulence by gadofosveset. Comparison of 3D SSFP with conventional MR sequences are shown in a bar graph (Figure 1). Mean duration for 3D SSFP was 5.12±0.48 min compared to 28.02±6.73 min for multiplanar 2D SSFP.

## Conclusions

RT 3D cine SSFP can replace conventional MR sequences for LV volumetry and function, aortic root measurement, and vessel diameter. Improved hardware and software, and improved spatial resolution are needed before it can replace current sequences for evaluation of intracardiac morphology and extracardiac vasculature.

## Funding

n/a.

